# Sustained Levels of FGF2 Maintain Undifferentiated Stem Cell Cultures with Biweekly Feeding

**DOI:** 10.1371/journal.pone.0056289

**Published:** 2013-02-20

**Authors:** Steven Lotz, Susan Goderie, Nicolas Tokas, Sarah E. Hirsch, Faizzan Ahmad, Barbara Corneo, Sheila Le, Akhilesh Banerjee, Ravi S. Kane, Jeffrey H. Stern, Sally Temple, Christopher A. Fasano

**Affiliations:** 1 Neural Stem Cell Institute, Rensselaer, New York, United States of America; 2 Department of Chemical and Biological Engineering, Rensselaer Polytechnic Institute, Troy, New York, United States of America; 3 New York State Department of Health, Department of Biomedical Sciences, State University of New York at Albany School of Public Health, Albany, New York, United States of America; Universidade de São Paulo, Brazil

## Abstract

An essential aspect of stem cell culture is the successful maintenance of the undifferentiated state. Many types of stem cells are FGF2 dependent, and pluripotent stem cells are maintained by replacing FGF2-containing media daily, while tissue-specific stem cells are typically fed every 3rd day. Frequent feeding, however, results in significant variation in growth factor levels due to FGF2 instability, which limits effective maintenance due to spontaneous differentiation. We report that stabilization of FGF2 levels using controlled release PLGA microspheres improves expression of stem cell markers, increases stem cell numbers and decreases spontaneous differentiation. The controlled release FGF2 additive reduces the frequency of media changes needed to maintain stem cell cultures, so that human embryonic stem cells and induced pluripotent stem cells can be maintained successfully with biweekly feedings.

## Introduction

Stem cells possess two hallmark properties: self-renewal and the ability to differentiate into one or more mature cell lineages. Most uses of stem cells involve first a period of culture in conditions that promote self-renewal to increase the number of stem cells, then a subsequent period of culture in distinct conditions that promote differentiation. The successful maintenance of stem cell cultures preserves the process of self-renewal while minimizing spontaneous differentiation into other cell types and, importantly, minimizing differentiative changes within individual stem cells that erode their pluripotency or multipotency. Optimizing methods to maintain stem cells is important to increase stem cell culture homogeneity, the number of stem cells produced and the potential to subsequently differentiate into lineages of choice. In vivo, stem cells are maintained in niches that control the growth factor environment to propagate the stem cell state [1], [2]. Here we have used this concept of stabilizing the growth factor environment to create an improved method for maintenance of stem cell cultures.

Current protocols for stem cell maintenance involve frequent feeding with growth factor-containing medium. For example, standard culture methods to maintain undifferentiated pluripotent stem cell cultures require daily replacement of the culture medium, making the care of these cells costly and labor intensive. Importantly, daily medium changes greatly reduce but do not fully eliminate spontaneous differentiation of pluripotent stem cell cultures [3], [4], [5], [6], [7], which leads to a gradual loss of potency and, often, to premature termination of the cultures. Fibroblast growth factor 2 (FGF2 or basic FGF) is a critical medium component for maintenance of a number of stem cell types, including human pluripotent stem cells. FGF2 has been reported to be highly labile at 37°C [8], [9], and we confirm dramatic fluctuations in FGF2 levels in standard stem cell culture protocols. Based on this, we hypothesized that fluctuations in growth factor levels are responsible for compromised stem cell maintenance, and we have found that sustained levels of FGF2 improve the in vitro maintenance of human pluripotent and neural stem cells.

## Results

### FGF2 Levels can be Stabilized in Stem Cell Cultures by Microsphere Encapsulation

**Figure 1:**
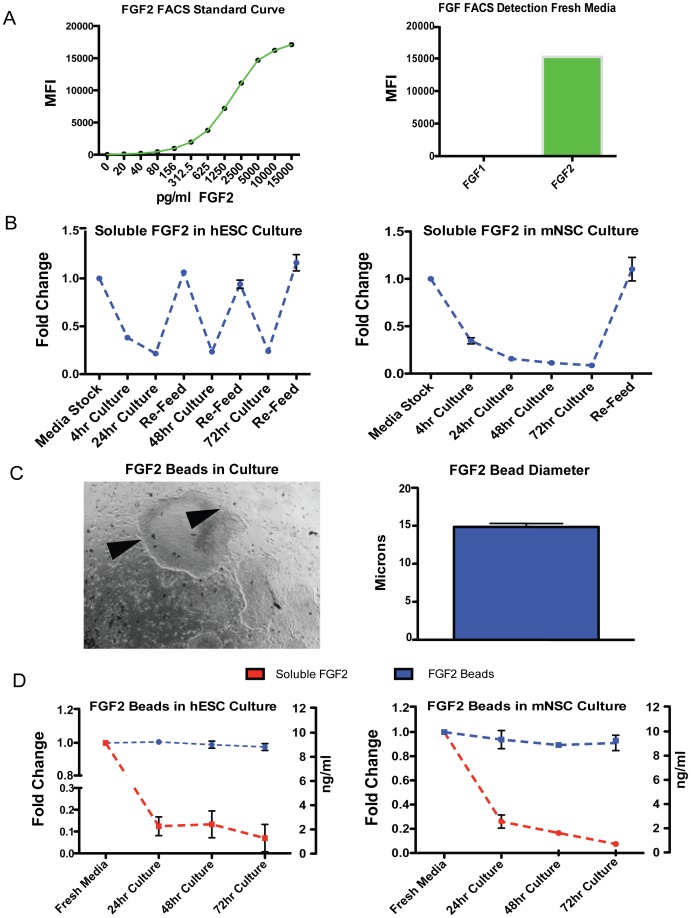
Sustained levels of FGF2 are achieved using PLGA microspheres. (A) A FACS based assay was used to measure FGF2 levels in culture. This method was sensitive at a range of concentrations and specific to FGF2. On the y-axis is Mean Fluorescence Intensity (MFI), the x-axis is concentrations of FGF2. (B) Standard daily medium changes in hESC culture lead to drastic fluctuations in FGF2 levels due to poor FGF2 stability. For mNSCs, the standard every 3rd day medium change leaves cultures with little FGF2 after the first 24 hours. (C) FGF2 beads visualized in cultures (arrows) have an average diameter of 14.5 microns scale bar = 100 microns. (D) Using PLGA microspheres, FGF2 is released at a constant rate over a 3 day period, sustaining FGF2 levels.

To investigate the stability of FGF2 in different stem cell culture media, stem cells were plated and FGF2 levels were measured over the course of three days using standard feeding protocols and a quantitative, flow-based assay (Fig. 1A). This assay uses a bead, coupled to an FGF2 antibody that can be added to stem cell culture medias to accurately measure the levels of FGF2 present. To ensure our method was measuring FGF2 specifically, we added FGF1 to cells in culture and using the same FGF2 antibody, we were unable to detect a signal suggesting the FGF2 antibody was specific (Fig. 1A). We found that just 4 hours after feeding, FGF2 levels had already decreased by more than 50% and 24 hours after the initial feed, little FGF2 remained. Upon re-feeding, the FGF2 levels spiked up, and this process was repeated daily, creating a highly unstable environment due to dramatic fluctuations in FGF2 levels that included significant periods of low FGF2 concentrations (Fig. 1B). Similar results were obtained when culturing mouse neural stem cells (mNSCs), using standard feeding protocols, but as these cells are typically fed every 3rd day, they had negligible FGF2 for almost two days (Fig. 1B). While FGF2 signaling can be improved by addition of heparin, this does not prevent the daily degradation of FGF2 [10]–[11] (data not shown). These observations led us to explore the effect of growing ESCs and NSCs in the presence of polymer-stabilized FGF2 levels.

Stable levels of FGF2 were produced using methods described previously for the controlled release of growth factors [12], [13]. We chose to encapsulate FGF2 in polyesters of glycolic and lactic acids (PLGA) which are biocompatible and have been developed to deliver protein drugs to patients [14]. Moreover, PLGA millicylinders that successfully encapsulate recombinant human FGF2 have been described [13]. Such PLGA complexes have been used to deliver growth factors during differentiation protocols to optimize production of mature progeny, for example to differentiate stem cells into osteoblast and chondrocyte-like cells [15], [16] but have not been used previously to maintain cultures of undifferentiated human pluripotent or other stem cell types. We modified the published protocols to encapsulate FGF2 with heparin for controlled release to maintain a concentration of 10 ng/ml in ESC and NSC culture media. The resulting FGF2 microspheres (hereafter termed FGF2 beads) range in size from 10.8–20.5 microns, with a mean diameter of 14.5 microns (Fig. 1C). When added to hESC medium or NSC medium at 37°C, the FGF2 beads maintained stable FGF2 levels for at least 3–4 days, much longer than measured using soluble FGF2 (Fig. 1D) It is important to note that while heparin is added to the FGF2 bead formulation and helps to achieve a constant, stable FGF2 level, it is not necessary for stable FGF2 levels (data not shown).

### Sustained FGF2 Better Maintains the Undifferentiated State with Less Frequent Media Changes in hPSCs

**Figure 2:**
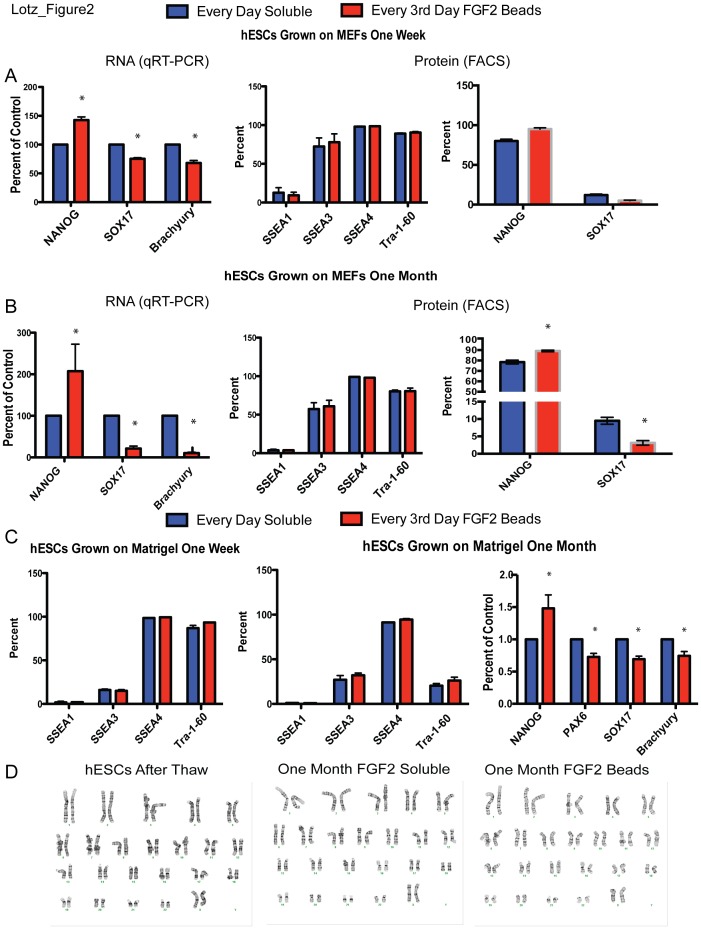
Culture with FGF2 beads and biweekly feeding produces a more undifferentiated hESC culture than traditional daily feeding. (A) hESCs grown one week on MEF feeders exhibit a similar FACS stem cell profile when fed with FGF2beads in medium every third day compared to soluble FGF2 in medium daily. However, the expression of Nanog was significantly increased and the differentiation markers SOX17 and Brachyury were significantly reduced, by qRT-PCR (left panel). (B) A similar but more pronounced effect was seen for hESCs grown for one month. (C) hESCs grown one week on matrigel exhibit a similar FACS stem cell profile when fed with medium+FGF2 beads every third day compared to medium+soluble FGF2 added daily. A similar effect was seen for hESCs grown for one month. After one month of growth, the pluripotency markers OCT4 and Nanog can be visualised by immunocytochemisty in both conditions. In addition, the expression of differentiation markers PAX6, SOX17 and Brachyury were significantly less by qRT-PCR. (D) Normal Karyotypes for hESCs were assessed before expansion and then after one month of expansion in either soluble FGF2 or FGF2 beads, and neither showed abnormalities.

To test the hypothesis that sustained levels of FGF2 lead to an improved undifferentiated hESC culture, we assessed pluripotency and differentiation markers. hESCs were grown for one week in standard mouse embryonic fibroblast (MEF) feeder conditions with standard daily feeding (soluble FGF2 in fresh medium) or with FGF2 beads in fresh medium every 3rd day (biweekly). Using FACS analysis, we observed similar surface expression of the stem cell markers SSEA-3, SSEA4 and Tra-1-60 and of the differentiation marker SSEA-1 (Fig. 2A). However, when we analyzed the marker of pluripotency NANOG, and the mesodermal and endodermal markers SOX17 and Brachyury, respectively, we found significant differences- FGF2 bead biweekly feeding resulted in increased NANOG expression and reduced spontaneous differentiation, indicated by lower SOX17 and Brachyury (Fig. 2A–B) compared to standard daily feeding. This was verified at the level of protein using intracellular FACS for NANOG and SOX17 (Figure 2A).

Longer-term growth was assessed for hESCs on MEFs, comparing standard daily feeding regimes to biweekly FGF2 bead feeding. At 1 month, FACS analysis revealed no significant difference in the stem cell markers SSEA-3, SSEA4, and Tra-1-60 or in the differentiation marker SSEA-1 (Fig. 2B). Cells in both conditions had a similar appearance and expressed OCT4 and NANOG in the nucleus (Figure S1), and again, levels of spontaneous differentiation were significantly reduced by growth with FGF2 beads compared to soluble FGF2 daily feeding, as indicated by SOX17 and Brachyury levels (Figure 2B). This was verified at the level of protein using intracellular FACS for NANOG and SOX17 (Figure 2B). Similar results were obtained when hESCs were grown in feeder free conditions using mTesr1 medium (Fig. 2C **)**. Importantly, expansion using FGF2 beads did not result in karyotypic abnormalities (Fig. 2D). We have grown hESCs for 14 passages in medium containing FGF2 beads with similar results (data not shown).

### Sustained FGF2 does not Alter the Differentiation Potential

**Figure 3:**
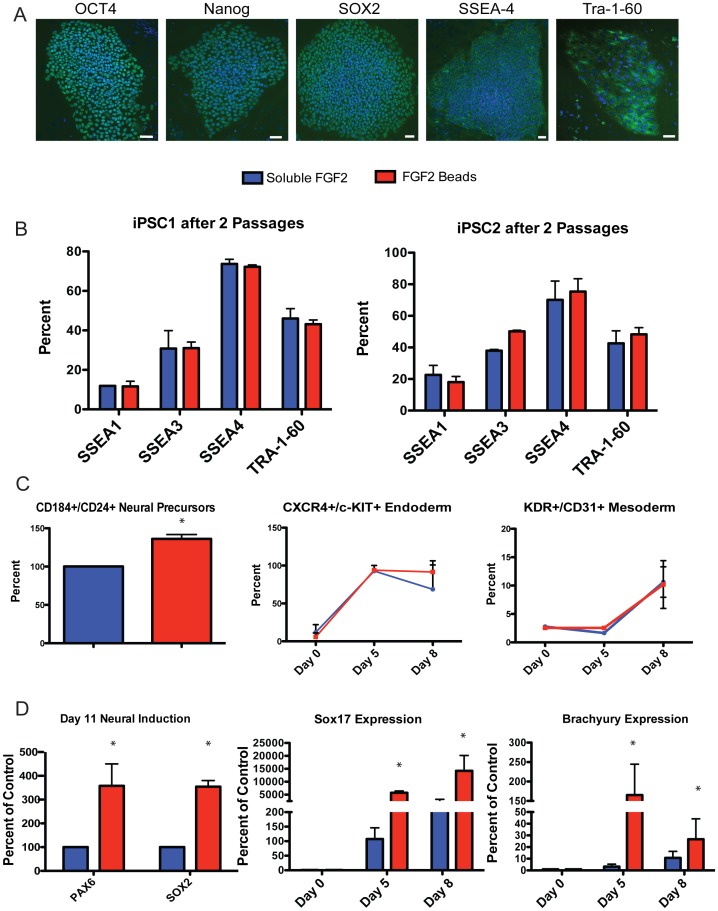
FGF2 maintain iPSC and increase differentiation potential of hESC. (A) iPSC lines were derived and assessed for pluripotency markers (B) iPSCs grown for two weeks on matrigel exhibit a similar FACS stem cell profile when fed with medium+FGF2 beads every third day compared to conventional medium+soluble FGF2 daily feeding. Scale bar = 50 microns. (C) Using directed differentiation protocols, hESC differentiation potential was assessed for all three germ lineages. From left to right panels, hESCs grown using FGF2 beads gave rise to neural, mesodermal, and endodermal progeny, assessed by FACS for relevant markers. (D) By qRT-PCR, FGF2 Beads expanded hESCs expressed higher levels of lineage markers after differentiation to all lineages.

Induced pluripotent stem cells (iPSCs) possess similar characteristics to hESCs and are also typically grown in culture conditions requiring daily addition of FGF2. Two iPSC lines were cultured with either soluble FGF2 (daily feeding) or FGF2 beads (biweekly feeding) for two passages and the FGF2 beads improved undifferentiated growth, similar to the results observed for hESCs (Fig. 3A–B). Collectively, these data demonstrate that sustained levels of FGF2 improve maintenance of undifferentiated pluripotent stem cells.

It was important to assess the differentiation capability of hESCs grown in the presence of FGF2 beads to determine the effect of a sustained FGF2 environment on the ability of these cells to produce differentiated progeny. hESCs were grown for 5 weeks in soluble FGF2 with daily feeding or FGF2 bead conditions with biweekly feeding, and then subjected to standard, directed tri-lineage differentiation protocols [17], [18], [19]. Efficient differentiation into all three germ layers was observed for both the daily feed and FGF2 bead culture conditions, assessed using FACS based assays (Fig. 3C) [17], [19], [20]. Notably, hESCs grown in the FGF2 bead condition had a higher differentiation potential as indicated by PAX6/SOX2 (neural), SOX17 (endodermal) and Brachyury (mesoderm) expression (Fig. 3D). These data indicate that by some assays, growth of pluripotent stem cells in a stable FGF2 environment during the expansion phase increases the differentiation potential of the cells compared to standard, soluble FGF2 daily feeding.

### Sustained FGF2 Better Maintains Neural Stem Cells in the Undifferentiated State

**Figure 4:**
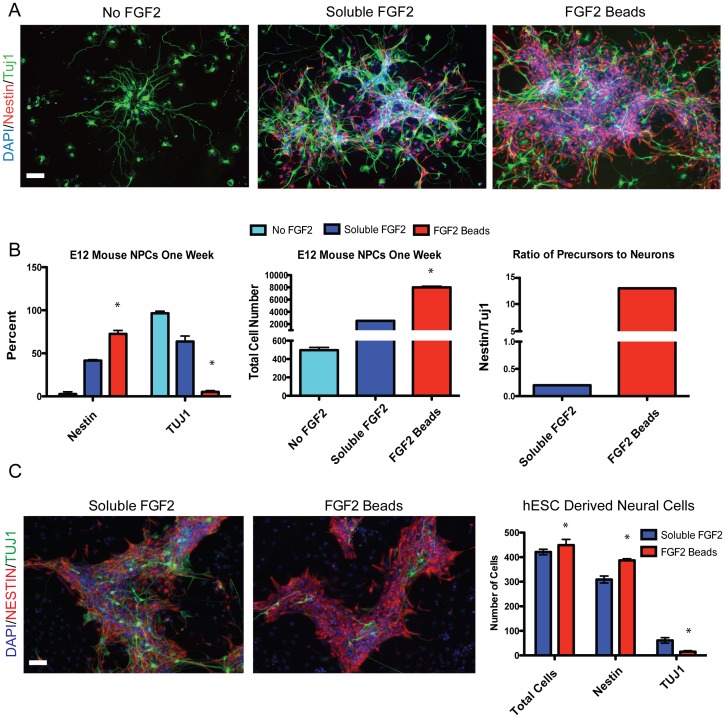
FGF2 beads produce a more undifferentiated, mouse and human neural stem cell culture. (A) Mouse NSCs grown for one week in FGF2beads show increased progenitor cells (Nestin+) and decreased neuronal differentiation (TUJ1+) compared to no FGF2 and soluble FGF2, data quantified in (B). (C) hESC-derived hNSCs grown for two weeks in the presence of FGF2 beads show higher levels of Nestin and lower levels of TUJ1 than those grown with soluble FGF2 (scale bars = 50 microns).

We next tested whether FGF2 beads had a similar effect on other stem cell types. Neural stem cells (NSCs) were isolated from embryonic mouse forebrain and three conditions were compared, each with biweekly (every third day) feeding: 1) Medium+No FGF2, 2) Medium+soluble FGF2 and 3) Medium+FGF2beads. After 7 days, the cultures were fixed for immunostaining and counting (Fig. 4A–B). In the first condition with no FGF2, little stem cell expansion was seen, few Nestin+ progenitor cells remained and most of the cells present had differentiated into TUJ1+ neurons. In the standard growth method with every third day feeding using soluble FGF2 (condition 2) a typical NSC culture was observed that included both Nestin+ neural progenitor cells and differentiated TUJ1+ neurons. In the third condition, with FGF2 beads added in medium every third day, the NSC number had markedly increased, by approximately 4-fold and the number of differentiated neurons had decreased, dramatically changing the ratio of neural precursor to neurons from 0.3 to 13.3 (Fig. 4A–B). This shows that sustained levels of FGF2 maintain NSC cultures more successfully than conventional soluble FGF2 feeding. Given the advantages for mouse NSC growth, we then assessed whether human NSCs could be maintained more successfully using FGF2 beads. Human NSCs (hNSCs) were derived from hESCs as previously described [18], plated at the same density, and fed with soluble FGF2 every 2 days or FGF2 beads biweekly. After two weeks, hNSCs grown using FGF2 beads showed a 28% increase in Nestin positive cells and a 46% decrease in spontaneous neuronal differentiation compared to the standard soluble FGF2 culture method (Fig. 4C). Hence, FGF2 beads can significantly increase the efficiency of hNSC growth and improve the homogeneity of NSC cultures.

### Sustained FGF2 Levels Better Activate the MAPK Pathway

**Figure 5:**
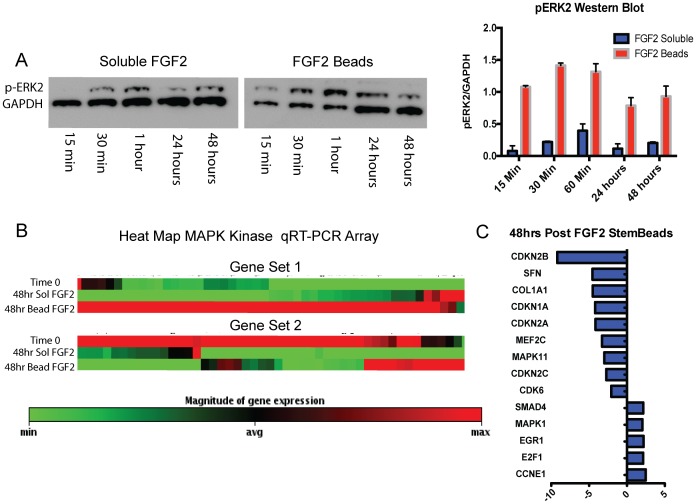
Sustained levels of FGF2 in stem cell culture improve activation of the MAPK pathway. (A) hESCs were fed with medium containing soluble FGF2 or FGF2 beads, 25 ugs of protein were loaded for each sample and pERK2 levels were assessed over the subsequent 48 hours by western blot (left panel), quantified by densitometry (right panel). FGF2 beads more efficiently activated the MAPK pathway, seen as early as 15 minutes post-addition, with activity still higher than baseline at 48 hours. (B) For hESCs, a global view of expression of 85 genes involved in the MAPK pathway reveals a very different signature in cells treated with FGF2 beads compared to soluble FGF2 at 48 hours. (C) qRT-PCR of specific genes in the pathway shows cell cycle inhibitors such as CDKN2B and CDKN2C were downregulated, while positive MAPK regulators MAPK1 and CCNE1 were upregulated in FGF2 bead culture compared to soluble FGF2. Genes listed were significantly different compared to soluble FGF2 at 48 hours.

FGF2 is known to activate the mitogen activated protein kinase (MAPK) pathway important for cell cycling and stem cell maintenance [21], [22], [23]. To test whether FGF2 beads impacted the MAPK pathway, we grew hESCs for 3 days and then added either soluble FGF2 (daily feeding in medium using standard methods) or FGF2 beads (added in medium once at the start of the experiment), and then followed MAPK activation over the next 48 hours. As early as 15 minutes after the start of the experiment, increased levels of p-ERK2 were observed in the FGF2 bead condition, indicating greater activation of the MAPK pathway compared to soluble FGF2 (Fig. 5A). This increase was maintained for the entire time-course. To further study the MAPK pathway response to FGF2 stimulation, we grew hESCs for 48 hours and isolated RNA at the beginning and end of the time-course. Control conditions used standard daily FGF2 medium feeding while experimental conditions used FGF2 beads in medium added once at the start of the experiment. An array of 85 genes known to be involved in the MAPK pathway were assayed using a MAPK qRT-PCR array. As indicated by the gene expression heat map, the profile for cells fed with routine soluble FGF2 was very different from that of cells fed once with FGF2 beads (Fig. 5B). We then examined the expression of particular genes in the MAPK pathway by qRT-PCR, focusing on the hESC cultures (Fig. 5C). Cell cycle inhibitors were expressed at significantly lower levels in the FGF2 bead condition compared to the daily soluble FGF2 condition. In contrast, positive regulators of the MAPK pathway such as c-FOS, MAPKs, CDKs, and cyclins were expressed at much higher levels with FGF2 beads (Fig. 5C). One gene of particular interest is MAPK11 or p38 which is required for trophoblastic differentiation of hESCs [24], [25], one of the first lineages to appear in hESC cultures during spontaneous differentiation [26], [27]. p38 is significantly down-regulated in the cells cultured with FGF2 beads compared to those in soluble FGF2 at 48 hours (Fig. 5C). These data suggest that FGF2 beads activate the MAPK pathway to promote growth, inhibit differentiation and more efficiently maintain pluripotent stem cell cultures than does standard soluble FGF2 daily feeding.

## Discussion

We described a novel method to culture stem cells in a stable FGF2 environment that results in increased stem cell markers, decreased differentiation markers and increased numbers of stem cells. This stable growth factor culture method builds on prior studies showing soluble growth factors, and notably FGF2, maintain stem cells in an undifferentiated state. The use of soluble growth factors to maintain stem cell cultures, however, requires frequent feeding presumably due to labile media components and even daily media replenishment does not completely inhibit spontaneous differentiation. The use of controlled release growth factor polymers avoids daily feeding of stem cell cultures, thereby reducing labor and the risks associated with manipulation of cultures. Furthermore, we were surprised at the extent to which sustained growth factor levels improved stem cell culture quality, decreased spontaneous differentiation and increased cell numbers.

An important part of our study is the ability of stem cells to maintain their differentiation potential after extended growth in sustained FGF2. While at the level of the RNA, we do see increases in marker expression after directed differentiation compared to soluble FGF2, at the level of the protein only neural differentiation seems to have a higher expression of markers. Interestingly, a recent study [28] showed that FGF2 directly represses PAX6 in hESCs. This group showed that when the FGF2 pathway is inhibited, NANOG is rapidly down regulated and PAX6 was up regulated and neural cells were generated. Because the FGF2 beads lead to a much more efficient MAPK activation, neural generating potential could be increased this way.

The requirement for daily or frequent change of traditional stem cell culture media initially suggested the presence of labile media components. We confirmed that FGF2 undergoes marked degradation in stem cell cultures (Figure 1). Alterations in FGF2 level are known to drive differentiation of some stem cell lineages [8], [29], [30], [31], [32]. This could, in part, explain why steady growth factor levels achieved by FGF2 beads both minimized spontaneous differentiation, and increased potency of cultures even when cultures were fed every third day. There may be labile media components other than FGF2 and stem cell cultures may also produce unwanted products that can accumulate over time, dependent on culture density, to limit culture stability beyond the 3–4 day feeding period we studied. These can be determined in future experiments and possibly addressed to further improve culture techniques.

The improvements in stem cell cultures we observed appear to be independent of the specific technique used to stabilize the FGF2 level. Frequent manual addition of FGF2, for example, produced results similar to controlled delivery using PLGA microspheres (data not shown). We anticipate that other biocompatible controlled release FGF2 media additives such as hydrogel or chitosan, would produce similar effects on stem cell cultures. Our working hypothesis is that any technique which provides stable FGF2 levels that resemble the normal in vivo niche activity more closely than unstable soluble FGF2 does, will also more effectively maintain stem cells in the culture dish.

Pluripotent stem cells are routinely grown on feeder cell layers such as mouse fibroblasts (MEFs) and/or in the presence of conditioned media to provide supplemental nutrients. Over the past few years, ‘feeder-free’ culture techniques have been developed [33]. These techniques utilize combinations of high FGF2 levels, biologically active substrates such as matrigel or laminin and/or manipulation of mitogens. Substrates and matrices have been modified to contain growth factors. Culture dish coatings such as matrigel [33] do not eliminate the need for daily media change of pluripotent stem cell cultures, presumably because FGF2 stabilization is not sufficient (Figure 3).

Matrix scaffolds that encapsulate stem cells to increase available surface area have also incorporated growth factor [34], [35], [36]. Stem cells encapsulated within such matrices grow well exposed to the growth factor microenvironment within the matrix scaffold. In contrast, the FGF2 beads are a simple media additive and do not encapsulate cells. Beads added to otherwise standard culture media and culture dish techniques release FGF2 to stabilize levels without a fundamental change such as cell encapsulation. As such, FGF2 beads provide a very simple new method to improve standard culture techniques for successfully maintaining undifferentiated stem cells.

## Methods

### Pluripotent Cells and Culture Conditions

hESCs (WA-09 WiCell; passages 32–50) and iPS lines (iPS-B49-1, iPS-B68-1; passages 4–10 see methods below on how they were made) were cultured on MEFs plated at 12–15,000 cells/cm2 (MEFs, Globalstem) in medium described previously [37]. For daily feeding, 10 ng/ml FGF-2 was added or for stable FGF2 conditions, 7.5 microliters of FGF2 beads (concentration shown to release 10 ng/ml) were added per 1 ml of growth media. Pluripotent cells grown without feeders were grown on Matrigel (Becton Dickinson) with either mTesR (Stem Cell Technologies) or MEF conditioned medium. Cells were passaged using 6 U/ml of dispase (Worthington) in hESC medium, washed and replated at a split of 1∶5 to 1∶10.

### iPSC Generation

Human fetal cornea fibroblasts from Advanced Bioscience Resource Inc., (gift of Dr. Sheldon Miller, NEI) were used as starting material for iPSCs generated by transduction with four retroviral supernatants containing OCT4, SOX2, KLF4 and c-MYC [38].The resulting iPSC lines were analyzed by immunostaining for expression of pluripotency markers, including OCT4 (Cell Signaling C30A3), SOX2 (Santa Cruz sc-17319), Tra-1-60 (BD 560173), Nanog (R&D AF1997), and SSEA4 (BD 560308) SSEA1 (BD 560127).

### Neural Induction

Feeder-free hESC neural induction was carried out as previously described [18]. Briefly, hESCs were plated at a density of 20,000 cells/cm^2^ on matrigel (BD) coated dishes in MEF conditioned hESC medium (CM) with 10 ng/mL of FGF-2 and10uM ROCK-inhibitor (Tocris). The ROCK inhibitor was withdrawn, and hESCs were allowed to expand in CM for 3 days or until they were nearly confluent and then differentiated with 10 nM TGF-β inhibitor (SB431542, Tocris) and 500 ng/mL of Noggin (R&D). Neural cells were removed with Accutase (Innovative) at day 11, 25,000 cells were plated in a 24 well plate.

### Directed Endoderm and Mesoderm Differentiation

Prior to differentiation, pluripotent cells were feeder-depleted and placed in suspension culture in the following conditions: For endoderm: serum-free media supplemented with 100 ng/ml activin A for 5 days [19] and mesoderm: StemPro34 (Invitrogen) media supplemented with 20 ng/ml BMP4 and 10 ng/ml FGF2 (D1–D3), then 10 ng/ml VEGF and 10 ng/ml FGF2 (D4–D6) [17]. Cells were collected and analyzed on a FACS Aria 2 Cell Sorter (BD Biosciences).

### Spontaneous Differentiation

hESCs were spontaneously differentiated by placing clumps of hESCs in 6-well tissue culture treated plates (Costar 3506) in hESC medium for 1 day to allow cells to settle on Matrigel (BD). This medium was changed to DMEM plus 10% FBS, with feeding every 2–4 days. After 2-weeks the cells were fixed in 4% paraformaldehyde (PFA, Santa Cruz Biotechnology) for 20 minutes and prepared for immunocytochemistry.

### Primary Neural Stem Cell and Neural Progenitor Cell Culture

NSCs and NPCs were isolated from the embryonic cortex as described previously [39]. Cells were grown in adherent conditions on PLL or PLO coated plates in neural medium containing B27 and N2 [37], [39], [40] and the presence of either 10 ng/ml FGF2 or FGF2 Beads.

### Microsphere Preparation

An aqueous solution containing human FGF2 (Miltenyi Bio) in PBS, magnesium hydroxide in TE Buffer and Heparin were added to a Dichloromethane (DCM) solution containing 200 µg of PLGA (75∶25) at a 1∶1 ratio. The solution was vortexed and then polyvinylalcohol (PVA) was added to produce a water/oil/water emulsion. It was then placed at 4 degrees overnight for DCM evaporation. The microspheres were then isolated by centrifugation (1500 rpm, 3 min) and washed 3X with distilled water. FGF2 StemBeads purchased from StemCulture LLC brought about similar effects.

### Quantitative Real-time PCR

Total RNA was extracted using an RNeasy kit (Qiagen). For each sample, 1 µg of total RNA was treated for DNA contamination and reverse transcribed using Quantitect (Qiagen). Amplified material was detected using Taqman probes (Life Technologies) and PCR mix (Kappa) on a Mastercycler RealPlex2 (Eppendorf). All results were normalized to an HPRT control and are from 3 technical replicates of 3 independent biological samples at each data point.

### Western Blot

Samples were collected and proteins were harvested using RIPA buffer. 3 biological replicates were used for all time points. Protein was quantified using a BCA assay and 20 µgs of protein were loaded in each well of a 4–12% Bis-Tris mini-gel (Invitrogen, NP0335Box) with MES buffer (Bio-Rad, #101–0789). Samples were transferred to a PVDF membrane (Invitrogen, LC2005) in Transfer buffer (Invitrogen, NP0006-1). Membranes were placed on a rocking shaker overnight at 4°C in staining buffer (TBS, 0.1% Tween, 3% BSA) and stained with pERK1/2 (1 µg/mL,Cell Signaling). Membranes were then stained with anti-rabbit IgG-HRP (Abcam, ab6721, 1∶3000) in staining buffer for 1 hour at room temperature. Finally, membranes were stained with WB luminol reagent (Santa Cruz Biotech, sc-2048) for one minute, and imaged on a ChemiDoc XRS (Bio-Rad, #170-8070).

### Immunostaining and FACS

FGF2 concentrations were measured using a cytokine bead array Flex set according to manufacturers protocol (BD). Cells were fixed in 4% paraformaldehyde, permeabilized with Triton-X100 in PBS and stained with primary antibodies visualized with appropriate fluorescently labeled secondary antibodies (Molecular Probes). Cultures were counterstained with the nuclear marker DAPI (Molecular Probes). Nanog (R&D 1∶100), OCT4 (Santa Cruz, 1∶500), PAX6 (DSHB, 1∶50), TUJ1 (Chemicon, 1∶500), Nestin (DSHB, 1∶200), SSEA1,SSEA-3, SSEA4, Tra-1-60 (Becton Dickinson). FGF2 levels were measured using a cytokine bead array (Becton Dickinson) according to instructions on a FACS Aria2.

### Statistical Analysis

Results shown are mean±s.e.m. Asterisks and # signs identify experimental groups that were significantly different from by one way or two way ANOVA with a Bonferroni correction for multiple comparisons (p-value, 0.05), where applicable. All experiments were performed with a minimum of n = 3.

## Supporting Information

Figure S1 **Sustained FGF2 Levels Maintain Pluripotent Marker Expression.** (A) Immunostaining of month old cultures shows similar appearance of colonies and expression of the pluripotency markers OCT4 and NANOG in both conditions, Scale = 50 microns.
(TIF)
