# Supplementary figures and images for: Sustained Levels of FGF2 Maintain Undifferentiated Stem Cell Cultures with Biweekly Feeding
Source: PLoS One. 2013 Feb 20;8(2):e56289. doi: 10.1371/journal.pone.0056289 (PMC3577833; doi:10.1371/journal.pone.0056289)

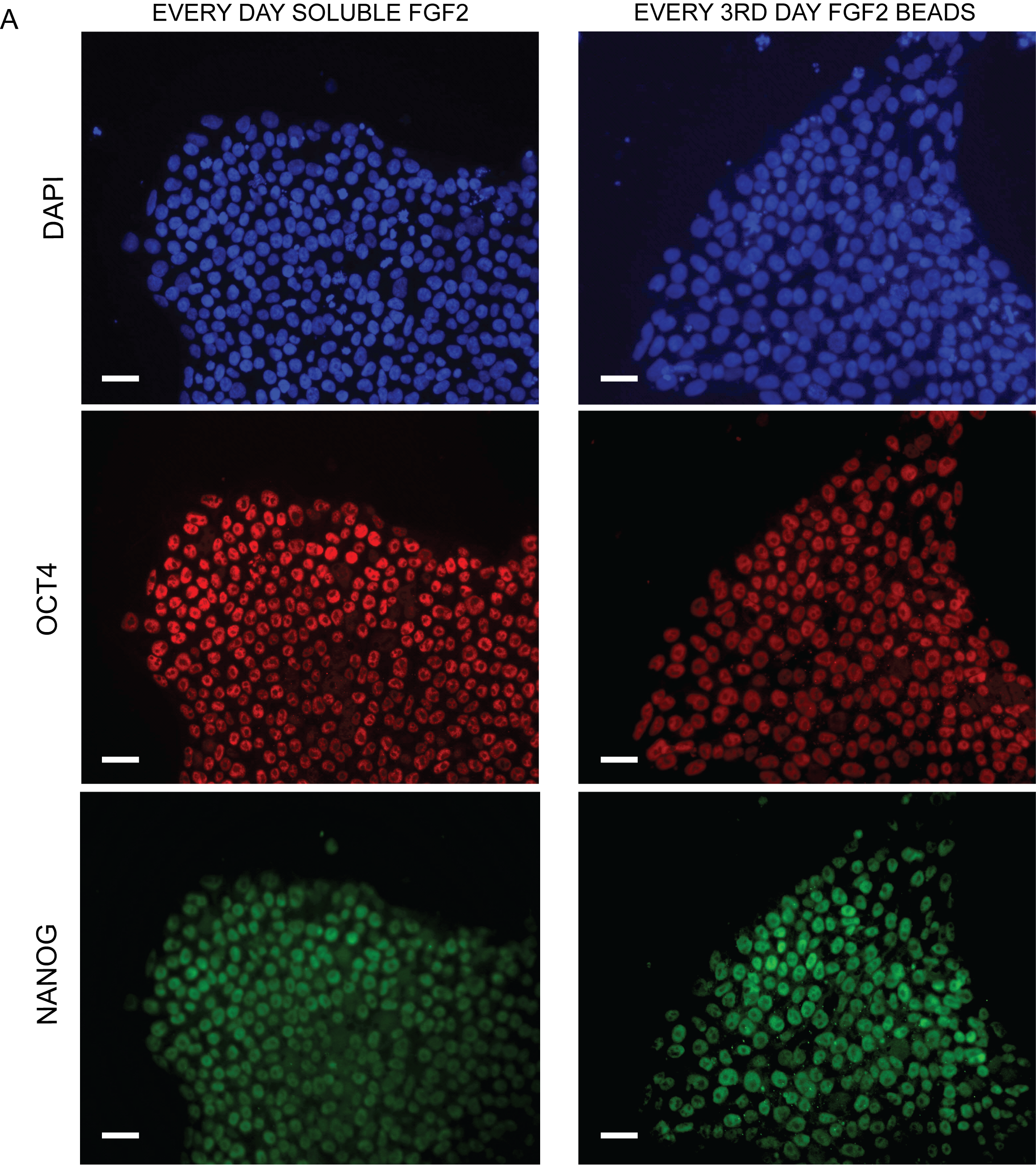

Supplement: Figure S1 — Sustained FGF2 Levels Maintain Pluripotent Marker Expression. (A) Immunostaining of month old cultures shows similar appearance of colonies and expression of the pluripotency markers OCT4 and NANOG in both conditions, Scale = 50 microns. (TIF) [file pone.0056289.s001.tif]
